# Scrub Typhus Outbreak in a Remote Primary School, Bhutan, 2014

**DOI:** 10.3201/eid2308.162021

**Published:** 2017-08

**Authors:** Tshokey Tshokey, Stephen Graves, Dorji Tshering, Kelzang Phuntsho, Karchung Tshering, John Stenos

**Affiliations:** Jigme Dorji Wangchuck National Referral Hospital, Thimphu, Bhutan (T. Tshokey);; University of Newcastle, Australia (T. Tshokey, S. Graves);; Australian Rickettsial Reference Laboratory (T. Tshokey, S. Graves, J. Stenos);; Bajo Hospital, Wangduephodrang, Bhutan (D. Tshering);; Royal Centre for Disease Control, Ministry of Health, Thimphu (K. Phuntsho, K. Tshering)

**Keywords:** Bhutan, scrub typhus, *Orientia tsutsugamushi* eschar, meningoencephalitis, thrombocytopenia, tsutsugamushi triangle, pediatric, vector-borne infections, chigger, larval mite, conjunctival congestion, myalgia, lymphadenitis, rash, eschar, parasites, encephalitis/meningitis

## Abstract

Scrub typhus in Bhutan was first reported in 2009. We investigated an outbreak of scrub typhus in a remote primary school during August–October 2014. Delay in recognition and treatment resulted in 2 deaths from meningoencephalitis. Scrub typhus warrants urgent public health interventions in Bhutan.

Scrub typhus, caused by the intracellular parasite *Orientia tsutsugamushi*, is a miteborne infection that largely occurs in the “tsutsugamushi triangle” (Technical Appendix Figure 1), where Watt et al. estimated ≈1 million cases occurred annually in 2003 (*1*). Infected persons commonly have fever, headache, conjunctival congestion, myalgia, lymphadenitis, rashes, and eschars with and without complications (*2*). Among untreated persons, the case-fatality rate is 6%–35% (*3*). In scrub typhus–endemic areas, central nervous system involvement occurs in ≈25% of patients (*4*). Consequently, scrub typhus should be considered in the differential diagnosis of aseptic meningitis. 

During January–October 2016, Nepal reported scrub typhus in 37 districts, resulting in 8 deaths (*5*). Himachal Pradesh, India, reported 700 case-patients, 20 of whom died (*6*). In Bhutan, scrub typhus gained clinical attention after an outbreak in 2009 (*7*); earlier cases might have been missed because of low awareness.

During August‒October 2014, a scrub typhus outbreak occurred in Singye Namgyal Primary School (SNPS), a remote community boarding school in the Wangduephodrang district of Bhutan (Technical Appendix Figure 2). On August 17, three girls from SNPS reported 5–6 days of fever, headache, cough, and body aches and were treated symptomatically by the visiting health assistant from Kamichu Basic Health Unit (KBHU). Two of the girls recovered; the third was admitted to the KBHU on August 20 and transferred to Bajo Hospital (BH) the next day. By August 26, she experienced neck stiffness, irritability, and disorientation. Viral encephalitis was suspected, and she was referred to the Jigme Dorji Wangchuck National Referral Hospital (JDWNRH) in Thimphu on August 27. On admission, a serum sample tested positive for *O. tsutsugamushi* by rapid diagnostic test (RDT); she died the next day. 

Another girl and her brother from SNPS were admitted to the Punakha district hospital on September 1 with similar symptoms. The boy was sent home with medications and recovered; his sister had meningeal symptoms and severe thrombocytopenia and was transferred to the JDWNRH on September 2, where she died on September 28. Specimens from both patients were *O. tsutsugamushi*–positive by RDT. 

On September 22, a 10-year-old girl from SNPS was referred to JDWNRH with similar symptoms. Her serum specimen was *O. tsutsugamushi* positive, but she recovered with treatment. 

After linking the 2 deaths and other cases, an investigation team visited the school during October 2–4. Case-patients were defined as any person from SNPS with fever, headache, and body ache with or without hemorrhagic manifestations currently or in the previous 2 weeks. Forty-one cases related to the outbreak were listed (Technical Appendix Figure 3); blood samples were drawn from 21 students, 12 of whom were acutely ill, and 10 local residents. Results for all 31 were negative for malaria and dengue; the Widal test of serum samples for enteric fever from 1 student and 2 local residents showed high antibody titers against *Salmonella* *enterica* serotype Typhi. Serum samples from the 12 acutely ill students were also tested for *O. tsutsugamushi* by RDT (SD Bioline Tsutsugamushi Test; Standard Diagnostics, Yongin, South Korea) in the Bhutan Public Health Laboratory; all were positive. The 12 samples were taken to the JDWNRH for routine blood tests; results showed anemia in 5 patients, thrombocytopenia in 4, neutropenia in 3, lymphocytosis in 2, and neutrophilia in 2 (Technical Appendix Table). The samples were also sent to the Australian Rickettsial Reference Laboratory (http://www.rickettsialab.org.au/), where they were tested for antibodies against *Orientia* by an indirect microimmunofluorescence assay (mIFA) (*8*). Of the 12 samples, 9 were positive (≥1:512 for IgM or ≥1:256 for IgG) (Table), including samples from all 6 students who had eschars. All samples from the 12 students were negative for *Orientia* DNA by using quantitative PCR.

**Table:** Antibody titers by indirect microimmunofluorescence assay of 9 students with diagnosis of scrub typhus, Bhutan, 2014*

Patient ID	Age, y/Sex	*Orientia tsutsugamushi*								*O. chuto*	
Gilliam			Karp			Kato	
IgG	IgM		IgG	IgM		IgG	IgM	IgG	IgM
2	6/M	256	512		256	512		256	128	<128	128
3	9/F	8,192	8,192		8,192	8,192		8,192	8,192	4,096	4,096
4	6/M	512	128		512	256		512	128	128	128
5	10/F	1,024	128		1,024	128		1,024	128	512	128
6	13/M	1,024	256		512	128		256	128	256	128
7	15/M	1,024	128		512	128		512	128	<128	<128
9	7/F	2,048	4,096		2,048	4,096		2,048	2,048	<128	<128
11	10/F	1,024	512		1,024	512		1,024	512	256	256
12	14/F	128	1,024		256	512		128	512	256	256

*ID, identification.

Of the acutely ill patients who had positive mIFA results, 67% had pathognomonic eschars, confirming the clinical diagnostic value in this sign of systemic infection. Thrombocytopenia as a sign of scrub typhus could be useful but is a less specific diagnostic indicator (*9*). There was only a 75% agreement between the rapid test kit and the precise mIFA, but RDTs were shown to be more useful in early detection (*10*)*.*

The deaths of 2 children in this outbreak could have been prevented if the public had greater awareness of the signs and symptoms of scrub typhus. Lapses of 7–10 days from symptom onset to initial medical consultation and >1 month until the outbreak was investigated demonstrate the importance of training school health coordinators to identify and report incidences of abnormal medical findings to public health agencies, especially in remote, hard-to-reach areas. Parents delayed seeking medical advice, and in some cases, school staff had to persuade them to take their children for medical evaluation. Rapid medical care during illnesses should be encouraged through better community education. 

Despite inadequate identification and reporting, there is increasing evidence of endemic scrub typhus in Bhutan. Outbreaks may be common but unrecognized, and past outbreaks may have been missed. Scrub typhus warrants a dedicated public health program or incorporation into the existing vectorborne disease control program in this country.

Technical Appendix Maps showing tsutsugamushi triangle and outbreak locations in Bhutan, and cases diagnosed during the outbreak.
