# Supplementary material for: Scrub Typhus Outbreak in a Remote Primary School, Bhutan, 2014
Source: Emerg Infect Dis. 2017 Aug;23(8):1412–4. doi: 10.3201/eid2308.162021 (PMC5547789; doi:10.3201/eid2308.162021)
Supplement: Technical Appendix — Maps showing tsutsugamushi triangle and outbreak locations in Bhutan, and cases diagnosed during the outbreak. [file 16-2021-Techapp-s1.pdf]

Article DOI: https://doi.org/10.3201/eid2308.162021

Scrub Typhus Outbreak in a Remote Primary School, Bhutan, 2014

Technical Appendix

Technical Appendix Table. Complete blood count of 12 acutely ill children infected with scrub typhus, Bhutan, 2014*

			Full blood count					
Pt. ID	Age (y)	Sex	**Total leucocyte count (10^3/μL) (NR=4–12)**	**Lymphocyte (%) (NR=20–50)**	**Neutrophil (%) (NR=40–65)**	**Platelet (10^3/μL) (NR=150–450)**	**Haemoglobin (g/dL) (NR=11–16)**	**Haematocrit (%) (NR=33–45)**
1	7	F	9.5	46	40	298	10.2	25
2†	6	M	6.5	47	39	272	10.0	30
3†	9	F	9.4	30	56	329	11.2	35
4†	6	M	8.1	34	45	169	12.0	36
5†	10	F	12.8	28	56	251	11.3	35
6†	13	M	4.5	21	70	121	13.2	41
7†	15	M	6.0	22	68	137	12.7	39
8	14	M	7.1	43	43	187	12.5	41
9†	7	F	7.9	31	58	92	12.2	35
10	8	M	4.6	57	31	193	9.3	29
11†	10	F	5.0	58	28	76	8.4	27
12†	14	F	11.4	32	59	209	9.9	30

*NR, normal range, Pt. ID, patient identification.
†Positive for scrub typhus by microimmunoflorescence.

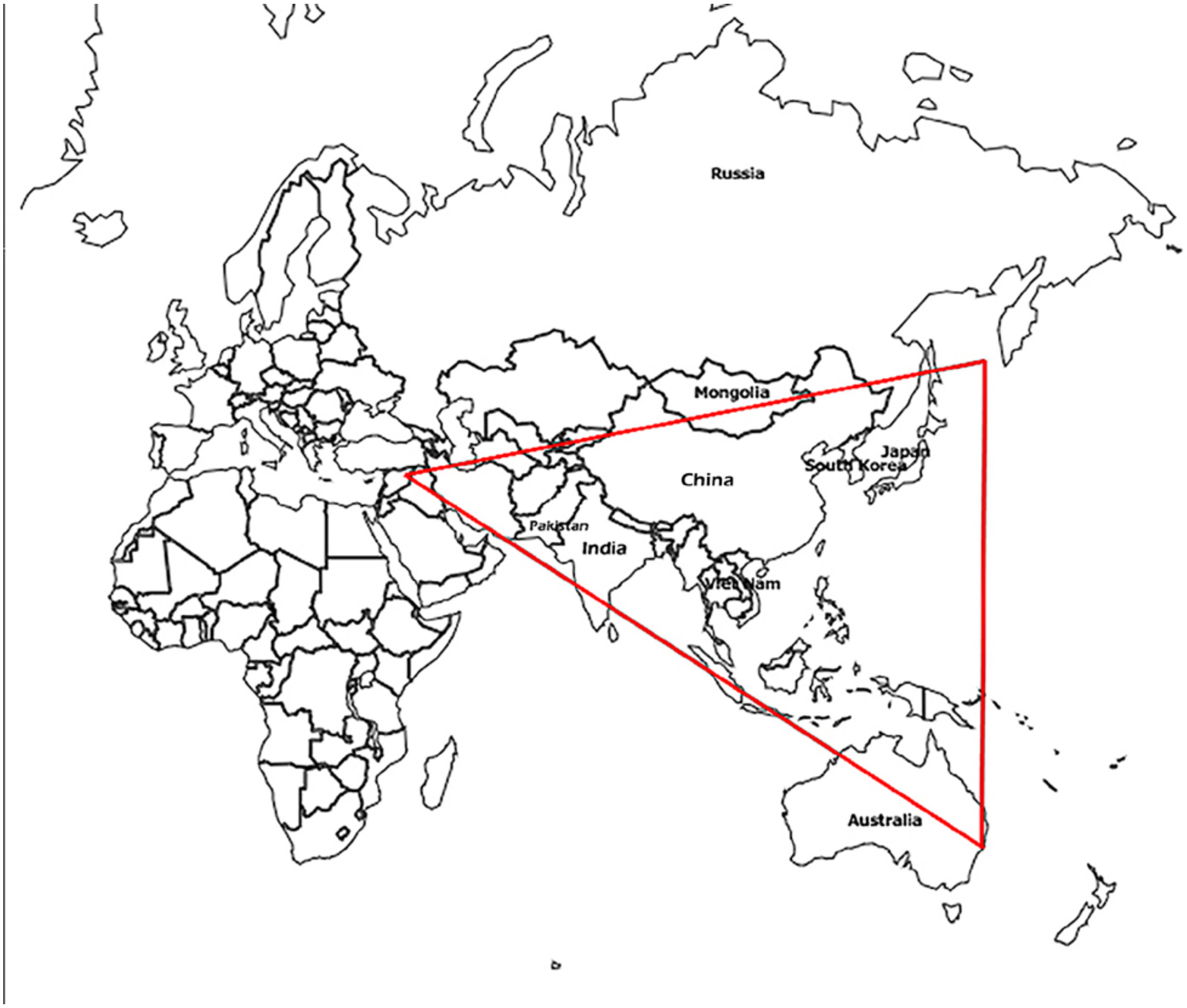

Technical Appendix Figure 1. The tsutsugamushi triangle (in red). More than 1,000,000 cases of scrub typhus, which is a mite-borne infection caused by the bacterium, *Orientia tsutsugamushi*, were reported in this area during 2003.

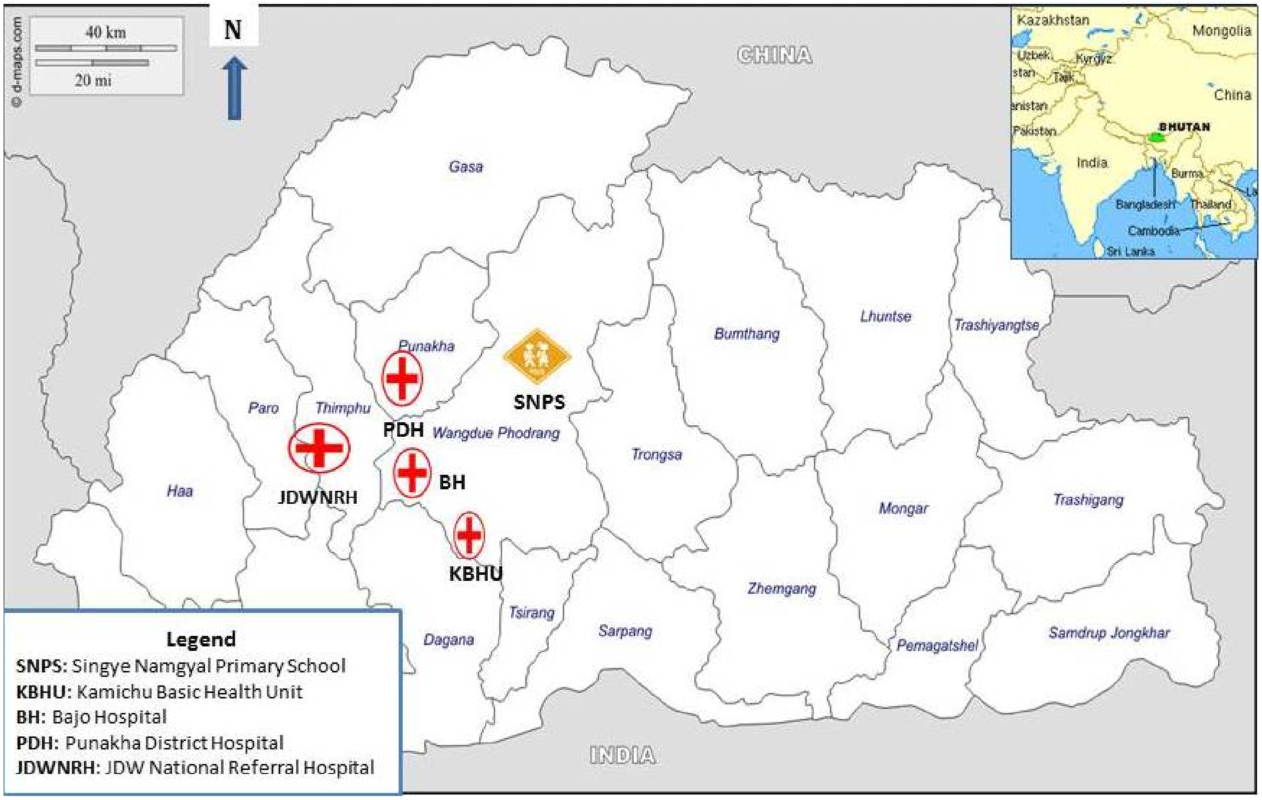

Technical Appendix Figure 2. Map of Bhutan showing the school and health centers relevant to outbreak of scrub typhus.

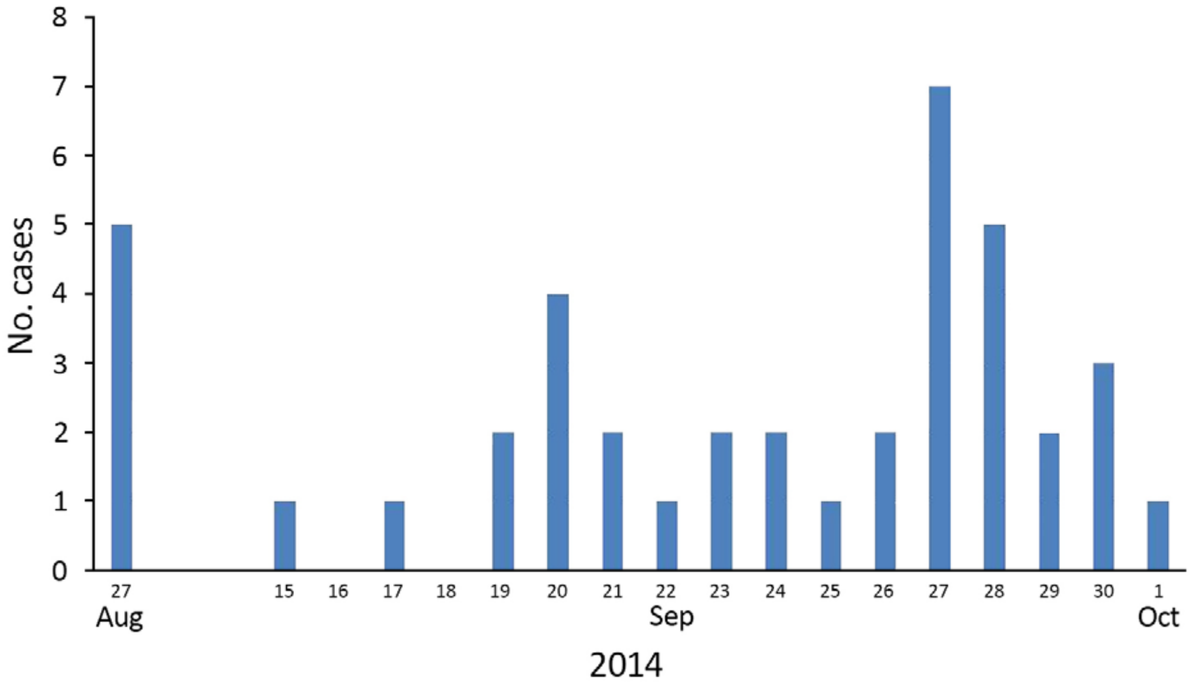

Technical Appendix Figure 3. Clinical cases of scrub typhus identified among students of Singye Namgyal Primary School in the Wangduephodrang district of Bhutan during August 27–October 1, 2014. Symptom onset among the first 3 case-patients began 5–6 days before being reported on August 17; the first incidence of scrub typhus in this cohort was diagnosed on August 27.
